# Bioequivalence and Pharmacokinetic Evaluation of 2 Pyrazinamide Formulations in Healthy Chinese Adults: A Single‐Dose, Open‐Label, Randomized‐Sequence, 2×2 Crossover Study

**DOI:** 10.1002/cpdd.1035

**Published:** 2021-11-16

**Authors:** Siyang Wang, Jian Ren, Hongxia Wang, Tingting Zhi, Yuanyuan Zhu, Jinxin Feng, Zhen Li, Ruiqin Zhang

**Affiliations:** ^1^ Department of Pharmacy Second Hospital of Shanxi Medical University Taiyuan China; ^2^ School of Pharmacy Shanxi Medical University Taiyuan China

**Keywords:** bioequivalence study, healthy subject, high‐performance liquid chromatography–tandem mass spectrometry, pharmacokinetic, pyrazinamide

## Abstract

A single‐dose, open‐label, randomized‐sequence, 2×2 crossover study was conducted in healthy Chinese adults, after fasting and postprandial, to evaluate the bioequivalence of 2 pyrazinamide (PZA) formulations. Fasting and postprandial tests were conducted in 24 cases. Test‐reference and reference‐test were randomly divided into 2 sequence groups, with 12 cases in each group. The concentration of PZA in plasma was determined after 0.5 g single oral PZA test and reference formulations by the high‐performance liquid chromatography–tandem mass spectrometry method. In the fasting group, the 90% confidence intervals (CIs) of the 2 formulations maximum plasma concentration (C_max_), area under the plasma concentration–time curve (AUC) from time 0 to last detectable plasma concentration, and AUC from time 0 to infinity after logarithmic conversion were 104.8% to 121.9%, 97.7% to 101.6%, and 97.7% to 101.6%, respectively. In the postprandial group, the 90%CIs of the 2 formulations’ C_max_, AUC from time 0 to last detectable plasma concentration, and AUC from time 0 to infinity after logarithmic conversion were 86.4% to 100.2%, 96% to 102%, 95.8% to 102.3%, respectively. The 90%CIs of the test/reference C_max_ ratio and AUC ratio were within the acceptable range of 80.00% to 125.00% for bioequivalence under both fasting and postprandial conditions. No serious adverse events occurred during treatment with the test formulation or the reference formulation.

Pyrazinamide (PZA), also known as isonicotinamide, is a nicotinamide structural analog with unique sterilization activity. Unlike other antituberculosis (anti‐TB) drugs, PZA can kill tuberculosis bacteria and retention bacteria in the semidormant period under acidic conditions. PZA has remarkable anti‐TB effect and can shorten the period of anti‐TB chemotherapy combined with other anti‐TB drugs, so it is recommended by the World Health Organization for the treatment of drug‐sensitive and multidrug‐resistant tuberculosis.

Previous studies have shown rapid absorption of PZA after oral administration, with peak serum concentrations achieved within 1 to 2 hours.^1^, ^2^, ^3^ The protein binding rate of PZA is very low, with a median of 1% in a study.^4^ PZA is extensively metabolized by the liver, and about 70% of drugs are excreted in the urine as metabolites. In adults with normal liver and kidney functions, the drug has an elimination half‐life of 8 to 11 hours.^5^ The bioavailability and peak serum concentration of PZA changes slightly when taken with food.^6^, ^7^

The aim of this study was to determine the blood concentration of PZA tablets in healthy Chinese adults and evaluate the bioequivalence of the 2 formulations, which provided a theoretical basis for drug registration and clinical trials.

## Study Design

### Drugs and Instruments

#### Drugs

PZA tablets (test formulation), specification: 0.5 g/tablet, manufacturer: Jiangsu Sihuan Biopharmaceutical Co., Ltd.

PZA tablets (reference formulation), specification: 0.5 g/tablet, manufacturer: DAVA PHARMACEUTICALS INC.

Instruments are shown in Table 1.

### Selection of Subjects

This study was conducted at the Phase I Clinical Trial Unit of the Second Hospital of Shanxi Medical University (Shanxi, China). The study was performed in accordance with the ethical principles for studies in humans described in the Declaration of Helsinki and its amendments, the International Conference on Harmonisation Guideline for Good Clinical Practice, and the Guideline for Good Clinical Principles recommended by the State Food and Drug Administration of China. The study protocol and the informed consent form were approved by the independent ethics committee of the Second Hospital of Shanxi Medical University. The study was registered with chinadrugtrials.org.cn (registry number: CTR20182436).

The enrolled subjects were required to meet the following inclusion criteria: age 18 to 45 years (inclusive); body mass index of 19.0 to 28.0 kg/m^2^ (inclusive); normal clinical and vital signs, 12‐lead electrocardiogram recordings and laboratory test results; and agreement to use effective contraception for at least 3 months after signing the informed consent form until the last drug administration of the trial.

The exclusion criteria were as follows: a clear history of diseases that could have interfered with the study results, and subjects with hyperuricemia or previous history. Detailed criteria are listed in Table 2.

### Grouping, Drug Administration, and Sample Collection

This was a single‐dose, open‐label, randomized‐sequence, 2×2 crossover study.

Fasting and postprandial tests were conducted in 24 cases. The participants were randomly divided into 2 sequence groups according to the test‐reference (T‐R) and reference‐test (R‐T) administration order, with 12 cases in each group. In the fasting group, after fasting for at least 10 hours, according to the random table, under the condition of fasting (postprandial group after high‐fat meal) from the morning of each cycle, 1 PZA tablet (test formulation) or 1 PZA tablet (reference formulation) was administered with 240 mL water. In the postprandial group, the same procedures were performed except that the participants consumed a high‐fat meal composed of 170 kcal of protein, 250 kcal of carbohydrates, and 480 kcal of fat before drug administration. PZA was administered exactly 30 minutes after the start of the high fat meals in the postprandial group.

Blood samples were collected at 0 minute (−60 minutes) before administration; 10, 20, 30, and 45 minutes; and 1, 1.5, 2, 2.5, 3, 4, 5, 6, 8, 12, 24, 36, and 48 hours after drug administration in each cycle. About 4 mL of venous blood was collected and placed in a dipotassium–ethylenediaminetetraacetic acid tube. Within 60 minutes after blood collection, the tubes were centrifuged at 2500 × *g*, at 2°Cto 8°C for 10 minutes to separate the plasma, and the plasma was aspirated into cryopreservation tubes and transferred to a −70°C ± 10°C freezer.

The study was conducted in 2 cycles, and all subjects needed a 7‐day washout period after the first cycle of drug administration before starting the next cycle. The overall flowchart of the process is shown in Figure S1.

### Method for Determining Sample Concentration

The human plasma samples were pretreated by protein precipitation, atomized ionization using electrospray ionization source, and the sample concentration was determined in a multireaction monitoring mode.

### Chromatography

Chromatographic column: Welch Ultimate LP‐C18 (2.1 × 100 mm, 3 μm; Welch, West Haven, Connecticut); mobile phase: 0.1% formic acid aqueous solution in A phase; 0.1% formic acid methanol solution in B phase; flow rate 0.4 mL/min; sample intake 10.0 μL.

### Mass Spectrometry

Mass spectrometry included a positive ion point spray ionization source, multireaction monitoring mode, ion spray voltage 4500 V, and ion source temperature 550°C.

### Plasma Sample Processing

We accurately transferred 50 μL of plasma samples in a 1.5 mL EP tube, and then added 200 μL of internal standard working solution (PZA‐d3 methanol solution with 250 ng/mL concentration), followed by vortexing for 3 minutes at 2500 rpm. The supernatant was centrifuged at 4°C and 12 000 × *g* for 5 minutes. Thereafter, 50 μL was added to each well of a 96‐well plate, followed by 150 μL methanol aqueous solution, vortexed for 5.0 minutes at 600 rpm, and mixed evenly. Then, a 10‐μL sample was analyzed by high‐performance liquid chromatography–tandem mass spectrometry.

### Methodological Evaluation

#### Selectivity

The blank conventional plasma from at least 6 different sources, blank hemolytic plasma from at least 3 different sources, and blank high‐fat plasma from at least 3 different sources were selected. Each plasma sample was 50 μL, and was processed according to “plasma sample processing.” The peak area of interference peak at the same retention time was less than 20.0% of the mean value of effective peak area in quantitative lower limit sample.

### Standard Curves and Quantitative Limits

The standard curve samples (50, 100, 300, 1000, 3000, 10 000, 22 500, 25 000 ng/mL) were taken and processed according to “plasma sample processing” for high‐performance liquid chromatography–tandem mass spectrometry analysis. The ratio and concentration of PZA peak area to internal standard peak area were regressed as PZA standard curve. The results showed an optimal linear relationship in the range of 50 to 25 000 ng/mL.

### Precision and Recovery

Human blank plasma was used as matrix, and 3 concentration quality control samples (150, 2000, and 20 000 ng/mL) were configured. Six parts per concentration were treated in parallel according to the “plasma sample processing,” and the recovery rate of matrix extraction was investigated. The precision coefficient of variation of the recovery rate of the object to be tested and the internal standard in the 3 concentration quality control samples was not more than 15% (8.27%, 12.06%, 8.56%); the total coefficient of variation of the recovery rate of the product to be tested and the internal standard was not more than 15.0% (9.82%, 9.47%).

### Matrix Effects

Human blank plasma from 6 different sources, human blank hemolysis plasma from 3 different sources, and human blank high‐fat plasma from 3 different sources were used to investigate the matrix effect of the low‐, medium‐, and high‐quality concentrations (150, 2000, and 20 000 ng/mL). The matrix effect factors of 3 concentrations of PZA (low, medium, and high) after internalization were 0.99, 1.00, and 0.99, while precision ( percent coefficient of variation) was 3.14, 2.30, and 2.21. After internal standard normalization, the total percent coefficient of variation of matrix effect factor was 2.61, which was <15%.

### Stability

The plasma samples were stable at room temperature for 24 hours, the treated samples were stable at 2°C to 8°C for 70 hours. The plasma samples were stable at −20°C and −70°C after freeze‐thawing for 4 times, and the plasma samples were stable at −20°C and −70°C for 44 days.

### Statistical Analysis

Statistical analysis of demographic characteristics and security was conducted using Windows System SAS (version 9.4; SAS Institute, Cary, North Carolina) software. Pharmacokinetic analysis was conducted with a noncompartmental method, using Phoenix WinNonlin version 7.0 (Pharsight Corp., Mountain View, California).

The maximum plasma concentration (C_max_) and the time to reach C_max_ (t_max_) were obtained directly from the concentration‐time curves. The area under the plasma concentration–time curve (AUC) from time 0 to last detectable plasma concentration (AUC_0‐t_) was calculated according to the linear trapezoidal rule. The AUC from time 0 to infinity (AUC_0‐∞_) was calculated as AUC_0–t_ + C_t_/λ_z_, where C_t_ was the last measured concentration and λ_z_ was the slope of the linear regression of the natural logarithm (ln)‐transformed concentration‐time curve. The elimination half‐life was calculated as 0.693/λ_z_. The apparent clearance after oral administration was calculated as dose/AUC_0‐∞_.

Descriptive statistics, including mean values and standard deviations, were used to summarize the pharmacokinetic data for the 2 drugs. An analysis of variance was performed on the ln‐transformed pharmacokinetic parameters (the AUC_0‐t_, AUC_0‐∞_, and C_max_), using the general linear model procedures in SAS. The analysis of variance model had fixed factors for sequence, treatment, period, and subject within sequence. The Wilcoxon signed‐rank test was used for nonparametric analysis to determine differences in the t_max_. If the 90% confidence intervals (CIs) of the AUC and C_max_ were within 80% to 125% of the statistical interval proposed by the Food and Drug Administration, the 2 drugs were considered to be bioequivalent.

## Results

### Demographic Data

Fasting and postprandial tests were conducted in 24 cases. T‐R and R‐T were randomly divided into 2 sequence groups, with 12 cases in each group. The demographic characteristics of the study population are summarized in Table S3.

### Blood Concentration–Time Curve

**Figure 1:**
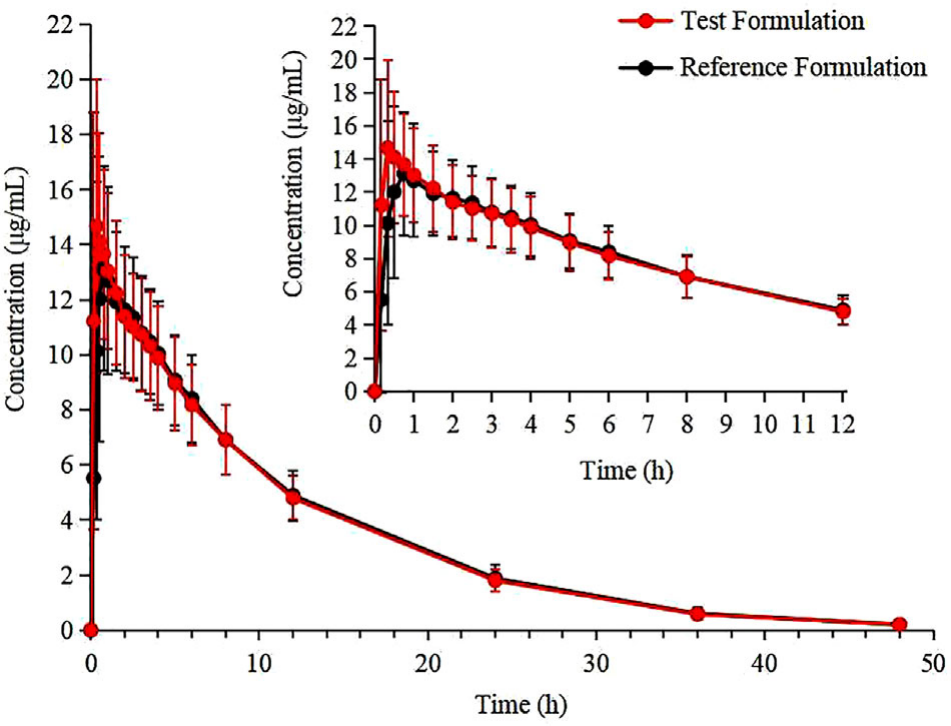
Mean (SD) plasma concentration‐time curve of 24 healthy subjects in the fasting group after administration of a single 0.5‐g dose of pyrazinamide tablet. All values below the quantification limit were recorded as 0 when calculating the mean values of plasma concentrations. All data are presented as mean with standard deviation.

The mean plasma concentration–time curve of 24 healthy subjects in the fasting group after administration of a single 0.5‐g tablet of PZA is shown in Figure 1.

**Figure 2:**
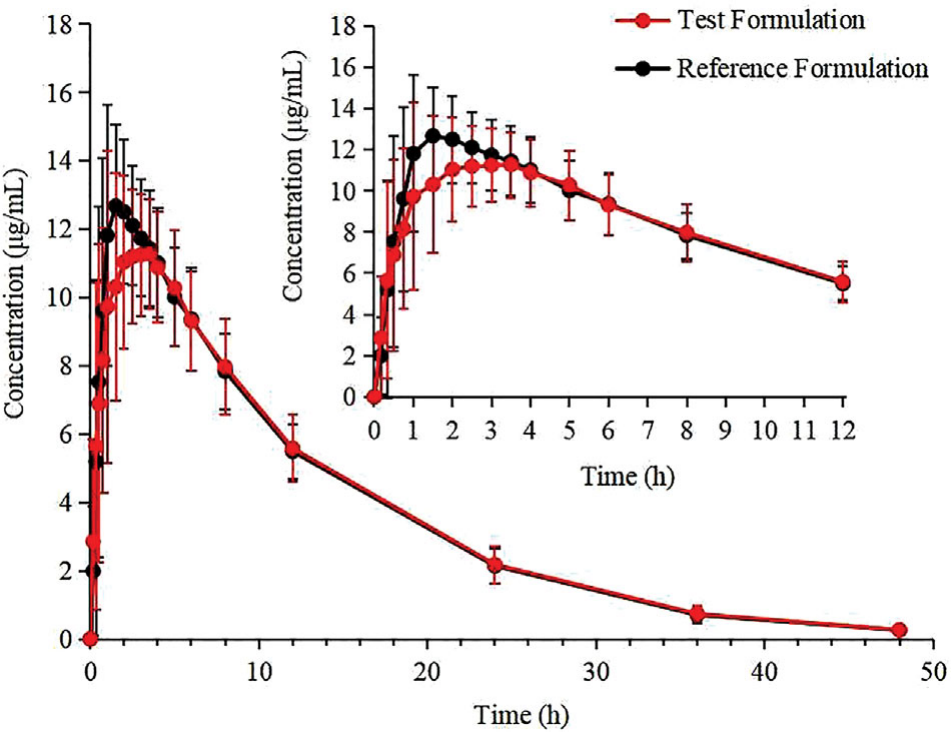
Mean plasma concentration‐time curve of 24 healthy subjects in the postprandial group after administration of a single 0.5‐g dose of pyrazinamide tablet. All values below the quantification limit were recorded as 0 when calculating the mean values of plasma concentrations. All data are presented as mean with standard deviation.

The mean plasma concentration–time curve of 24 healthy subjects in the postprandial group after administration of a single 0.5‐g tablet of PZA is shown in Figure 2.

### Pharmacokinetic Parameters

Table 1 lists the main pharmacokinetic parameters after administration of 0.5‐g PZA tablets. One subject in the T‐R sequence group used another medication during the washout period, and was excluded before the second cycle of administration, so there were 23 cases of oral reference formulation in the fasting group.

**Table 1:** Average PK Parameters After Administration of 0.5‐g Pyrazinamide Tablets

	Mean ± SD (Inter‐Individual %CV)			
	Fasting Group		Postprandial Group	
Parameters	Test (n = 24)	Reference (n = 23)	Test (n = 24)	Reference (n = 24)
t_max_,^a^ h	0.4 (0.2, 3.0) (97.6)	0.8 (0.3, 2.5) (69.0)	2.0 (0.3, 5.0) (60.8)	1.5 (0.3, 5.0) (58.8)
C_max_, μg/mL	16.8 ± 4.4 (26.5)	15.0 ± 4.5 (30.0)	13.4 ± 2.4 (17.7)	14.5 ± 2.9 (19.9)
AUC_0‐t_, μg/mL × h	161 ± 27 (16.7)	161 ± 29 (18.2)	174 ± 24 (14.1)	175 ± 22 (12.4)
AUC_0‐∞_, μg/mL × h	163 ± 27 (16.7)	164 ± 30 (18.2)	177 ± 25 (14.1)	178 ± 22 (12.5)
t_1/2_, h	7.7 ± 0.8 (10.2)	7.6 ± 1.0 (12.5)	7.9 ± 1.1 (13.3)	7.7 ± 1.3 (16.9)
CL/F, L/h	3.2 ± 0.6 (17.9)	3.2 ± 0.6 (18.4)	2.9 ± 0.4 (14.4)	2.9 ± 0.4 (13.2)

AUC, area under the plasma concentration–time curve; AUC_0‐∞_, AUC from time 0 to infinity; AUC_0‐t_, AUC from time 0 to last detectable plasma concentration; CL/F, apparent clearance; C_max_, maximum plasma concentration; CV, coefficient of variation; t_1/2_, elimination half‐life time; t_max_, time to reach C_max_.

^a^The median (minimum, maximum) is used. One subject in the fasting group T‐R sequence was use other medication at the washout period, which withdrew before the second cycle of administration.

### Bioequivalence Assessment

Evaluation results of bioequivalence after administration of 0.5‐g PZA tablets are shown in Table 2.

**Table 2:** Evaluation Results of Bioequivalence After Administration of 0.5‐g Pyrazinamide Tablets in 24 Subjects

	Geometric Mean and Ratio (N = 24)					
PK Parameters	Test	Reference	GMR, %	Intra‐Variation of Reference Formulation (CV%)	90%CI	Power Value, %
Fasting group						
Ln AUC_0–t_, μg • h/mL	159	159	1.0	3.8	97.7‐101.6	100
Ln AUC_0‐∞_, μg • h /mL	161	162	99.4	4.0	97.7‐101.6	100
Ln C_max_, μg/mL	16.2	14.3	113.3	15.0	104.8‐121.9	72.0
Postprandial group						
Ln AUC_0–t_, μg • h/mL	172	174	98.9	6.2	96.0‐102.0	100
Ln AUC_0–∞_, μg • h/mL	175	177	98.9	6.6	95.8‐102.3	100
Ln C_max_, μg/mL	13.2	14.2	93.0	14.9	86.4‐100.2	96.1

CI, confidence interval; GMR, geometric mean ratio; Ln AUC_0‐t_, area under the plasma concentration–time curve from time 0 to last detectable plasma concentration after logarithmic conversion; Ln AUC_0–∞_, area under the plasma concentration–time curve from time 0 to infinity after logarithmic conversion; Ln C_max_, C_max_ after logarithmic conversion.

### Safety Evaluation

The tolerability of the 2 formulations of PZA, each as a single administration, was acceptable. No serious adverse events (AEs) occurred during treatment with the test formulation or the reference formulation.

In the fasting group, a total of 14 AEs were observed in 24 subjects during the study, and the severity of all AEs was mild. Three AEs (1 abdominalgia for test formulation; and 1 abdominalgia, 1 hyperuricemia for reference formulation) were considered to be probably related to the study medication.

In the postprandial group, a total of 3 AEs were observed in 24 subjects during the study, and the severity of all AEs was mild. No AEs were considered to be related to the study drug. All AEs are listed in Table S4.

## Discussion

This study assessed the bioequivalence of a fasting and postprandial single dose test and reference formulations of 0.5 g oral PZA tablets in healthy Chinese adults. As shown in Figures 1 and 2, the 2 formulations had similar blood drug concentration–time curves. After logarithmic conversion, 90% confidence intervals of pharmacokinetic parameters (Table 2) including the C_max_, AUC_0‐t_, and AUC0_0‐∞_ of the 2 formulations were in the 80% to 125% range, suggesting that the 2 formulations were bioequivalent.

Table 1 shows the main PK parameters under fasting and postprandial conditions. For the reference formulation, t_max_ was significantly prolonged after a high‐fat meal, suggesting that food may delay the absorption rate of PZA. C_max_, AUC_0‐t_, and AUC_0‐∞_ were similar between the 2 conditions, indicating that food has little effect on the extent of PZA. The result was similar in the group taking the test formulation. A high‐fat meal had little effect on the PK profiles of PZA in healthy Chinese subjects, which was consistent with a previous study.^7^

Meanwhile, the safety evaluation showed that PZA tablets were safe and well tolerated. There was no significant difference in the incidence and severity of AEs between the 2 formulations. All AEs were mild, and no unexpected and serious AEs occurred.

There are a few reports on the pharmacokinetics of PZA, but most of the reports did not evaluate the bioequivalence of PZA alone. In a safety, tolerability, and bioequivalence assessment of a dispersible tablet formulation of PZA,^8^ a total of 36 subjects were enrolled and the bioequivalence of 2 formulations were tested under fasting condition. The C_max_ and AUC_0‐t_ of reference formulation of PZA (0.5 g/tablet) were 13.925 μg/mL and 161.053 μg • h/mL , respectively, which were slightly lower than our study.

Although many studies evaluated the pharmacokinetic characteristics of PZA, they combined PZA with other anti‐TB drugs.^9^, ^10^ In an oral bioavailability study of rifampicin, isoniazid, ethambutol, and PZA in a 4‐drug fixed‐dose combination compared with the separate formulations in healthy Chinese male volunteers, the pharmacokinetic parameters of PZA were not described separately.^10^ Similarly, another bioequivalence study examined a fixed‐dose combination of Myrin‐P Forte and reference drugs in loose combination.^9^ Our study excluded the interference of other drugs and was able to independently evaluate the pharmacokinetics of PZA in healthy subjects, which provided a theoretical basis for clinical application.

## Conclusion

This study in healthy Chinese adults suggested that the test formulation met the regulatory criteria for bioequivalence compared to the reference formulation, on the basis of the rate and extent of absorption. Both formulations were well tolerated.

## Conflicts of Interest

The authors declare no conflicts of interest.

## Funding

The Second Hospital of Shanxi Medical University Doctor's Funds, Grant/Award Number: 201702‐03. The funder, Yuanyuan Zhu, determined plasma concentrations of pyrazinamide. The Natural Science Foundation of Shanxi Province, Grant/Award Number: 201801D21221; Scientific and Technological Innovation Programs of Higher Education Institutions in Shanxi, Grant/Award Number: 20190115. The funder of these 2 fundings, Ruiqin Zhang, is the corresponding author.

## Supporting information

SUPPLEMENTARY INFORMATION
