# Supplementary material for: Bioequivalence and Pharmacokinetic Evaluation of 2 Pyrazinamide Formulations in Healthy Chinese Adults: A Single‐Dose, Open‐Label, Randomized‐Sequence, 2×2 Crossover Study
Source: Clin Pharmacol Drug Dev. 2021 Nov 16;11(4):551–6. doi: 10.1002/cpdd.1035 (PMC9298828; doi:10.1002/cpdd.1035)
Supplement: Supplementary file 1 — SUPPLEMENTARY INFORMATION [file CPDD-11-551-s001.docx]

**Supplemental Figure S1. Overall flow chart of bioequivalence evaluation of two pyrazinamide formulations in healthy Chinese adults**

Test Formulation: Pyrazinamide tablets, specification: 0.5 g/tablet, manufacturer: Jiangsu Sihuan Biopharmaceutical Co., Ltd.

Reference Formulation: Pyrazinamide tablets, specification: 0.5 g/tablet, manufacturer: DAVA PHARMACEUTICALS INC.

**
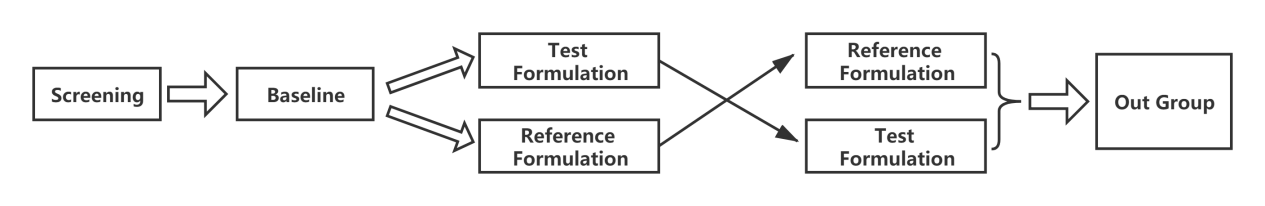
**

**Supplemental Table S1. Analytical instruments and components**

| Instruments | Model | Supplier |
| --- | --- | --- |
| Watson LIMS system | 7.5 SP1 | Thermo |
| AB SCIEX liquid-mass spectrometry | DGU-20A_5R_On-line degassing machine | SHIMADZU |
|  | Nexera X2LC-30AD dual high pressure pump |  |
|  | CTO-20AC temperature box |  |
|  | Nexera X2SIL-30AC automatic sampler |  |
|  | TRIPLE QUAD 4500 Triple Band 4 | SCIEX |
|  | Analyst 1.6.3 software |  |
| Mettler LabX 2017 System | XPE26 electronic balance | Mettler Toledo |
|  | XPE205 electronic balance |  |
|  | LabX 2017 software |  |
| Mixer | MX-S | SCILOGEX |
| Digital display multi-tube mixer | DVX-2500 | VWR |
| Pure water meter | Milli-Q reference ultrapure water system | Millipore |
| Low temperature high speed centrifuge | 3-18KS | Sigma |
| Water bath oscillator | SHZ-B | Spring orchid instrument |
| Pipette | * | Gilson/Eppendorft |

**Supplemental Table S2. Inclusion and exclusion criteria**

**Inclusion criteria:**

1) Chinese adults aged 18-45 years (including 18 and 45 years old).

2) The body mass index (BMI):19.0-28.0 kg/m^2^ (including 19.0 and 28.0).

3) The results of physical examination, vital signs, electrocardiogram and laboratory examination were normal, and the researchers judged that the subjects were in good health and mental state.

4) Subjects were willing to take appropriate contraceptive measures during the study period and within three months after withdrawal to avoid pregnancy or partner pregnancy.

5) The subjects were able to communicate fluently with the researchers, fully understand and strictly follow the research process, participate voluntarily, and sign informed consent.

**Exclusion criteria:**

1) Previously known or suspected to be allergic to pyrazinamide or its excipients, isoniazid, niacin, or other drugs with similar chemical structures, or with a severe allergic condition (known to be allergic to two or more different ingredients or to three or more food items).

2) Previous history of blood/needle phobia and intolerance to venous blood collection.

3) Subjects with hyperuricemia or previous history.

4) Subjects with clinically significant electrocardiogram abnormalities.

5) Subjects with severe systemic diseases or mental disorders.

6) Compounding diseases that may affect drug absorption, distribution, metabolism, excretion, and interpretation of safety data, or that may reduce compliance.

7) The results of hepatitis C antibody (HCV-Ab), Treponema pallidum antibody (anti-TP) and human immunodeficiency virus antibody (HIV-Ab) were positive.

8) The results of examinations of hepatitis B were determined by researchers to show clinical significance of liver disease.

9) Special diets, chocolate, caffeine, alcoholic beverages, or smoking, which may affect drug absorption, distribution, metabolism, excretion, could not be banned from 48 hours before each study period until the trial hospitalization.

10) Any drug or functional food used within two weeks before administration.

11) Any CYP450 inhibitors or CYP450 inducers used within four weeks before administration.

12) Smoking more than five cigarettes a day within three months before the study or could not stop smoking throughout the hospitalization period.

13) Regular drinkers of an average of more than two units of alcohol per day during the first three months of the study or alcohol expiratory test positive when entering the group.

14) Those who donated blood or lost blood ≥400 ml within three months before the study.

15) Those who participated in other clinical trials and used trial medicine or medical devices within three months before the study.

16) Those who had taken drugs within three months before the test or had a history of drug abuse within 12 months or were positive in urine drug screening tests.

17) Pregnant or lactating women.

18) Those with special dietary requirements.

19) The subjects were unable to complete the study or unsuitable participants due to other reasons.

**Supplemental Table S3. Baseline characteristics**

1. **Baseline characteristics of the study population in the fasting group**

| Characteristic |  | T-R Group | R-T Group | Total |
| --- | --- | --- | --- | --- |
| Age (years) | N | 12 | 12 | 24 |
|  | Mean(SD) | 24.3(2.9) | 23.8(2.6) | 24.0(2.7) |
|  | Median(Q1,Q3) | 23.5(23,26) | 24.0(22,26) | 24.0(22,26) |
|  | Min,Max | 21,32 | 19,28 | 19,32 |
| Height (cm) | N | 12 | 12 | 24 |
|  | Mean(SD) | 164.3(7.7) | 163.2(7.7) | 163.8(7.6) |
|  | Median(Q1,Q3) | 163.5(161,172) | 163.3(157,168) | 163.5(158,170) |
|  | Min,Max | 149,175 | 153,177 | 149,177 |
| Weight (kg) | N | 12 | 12 | 24 |
|  | Mean(SD) | 61.1(7.6) | 60.8(8.7) | 60.9(8.0) |
|  | Median(Q1,Q3) | 61.4(54,67) | 62.8(53,64) | 62.1(53,66) |
|  | Min,Max | 49,71 | 47,77 | 47,77 |
| BMI (kg/m^2^) | N | 12 | 12 | 24 |
|  | Mean(SD) | 22.6(1.9) | 22.8(2.3) | 22.7(2.1) |
|  | Median(Q1,Q3) | 22.8(21,24) | 22.9(21,25) | 22.9(21,24) |
|  | Min,Max | 19,25 | 20,26 | 19,26 |

1. **Baseline characteristics of the study population in the postprandial group**

| Characteristic |  | T-R Group | R-T Group | Total |
| --- | --- | --- | --- | --- |
| Age (years) | N | 12 | 12 | 24 |
|  | Mean(SD) | 28.3(5.3) | 26.2(5.1) | 27.3(5.2) |
|  | Median(Q1,Q3) | 26.5(26,30) | 25.0(24,26) | 26.0(25,27) |
|  | Min,Max | 23,43 | 22,42 | 22,43 |
| Height (cm) | N | 12 | 12 | 24 |
|  | Mean(SD) | 163.9(7.4) | 164.8(10.5) | 164.3(8.9) |
|  | Median(Q1,Q3) | 162.8(158,168) | 163.5(157,172) | 162.8(157,172) |
|  | Min,Max | 154,180 | 149,186 | 149,186 |
| Weight (kg) | N | 12 | 12 | 24 |
|  | Mean(SD) | 62.5(11.6) | 64.4(14.4) | 63.4(12.8) |
|  | Median(Q1,Q3) | 56.8(54,69) | 56.7(53,76) | 56.7(54,74) |
|  | Min,Max | 51,89 | 50,94 | 50,94 |
| BMI (kg/m^2^) | N | 12 | 12 | 24 |
|  | Mean(SD) | 23.1(2.2) | 23.5(2.8) | 23.3(2.5) |
|  | Median(Q1,Q3) | 22.7(21,24) | 23.1(21,26) | 22.9(21,25) |
|  | Min,Max | 20,28 | 19,28 | 19,28 |

**Supplemental Table S4. Adverse Events**

1. **AEs after administration of 0.5 g pyrazinamide tablets in the fasting group**

|  | Test formulation (N=24) | | Reference formulation (N=23) | | Overall (N=24) | |
| --- | --- | --- | --- | --- | --- | --- |
| Indicator | Subjects(%) | Cases | Subjects(%) | Cases | Subjects(%) | Cases |
| Laboratory examination | 4(16.67%) | 5 | 4(17.39%) | 4 | 7(29.17%) | 9 |
| Urine leucocyte positive | 2(8.33%) | 2 | 2(8.70%) | 2 | 4(16.67%) | 4 |
| Hyperuricemia | 0 | 0 | 1(4.35%) | 1 | 1(4.17%) | 1 |
| Decreased fibrinogen | 3(12.50%) | 3 | 1(4.35%) | 1 | 3(12.50%) | 4 |
| Dermatologic | 1(4.17%) | 1 | 0 | 0 | 1(4.17%) | 1 |
| Maculopapular rash | 1(4.17%) | 1 | 0 | 0 | 1(4.17%) | 1 |
| Gastrointestinal | 2(8.33%) | 2 | 1(4.35%) | 1 | 2(8.33%) | 3 |
| Abdominalgia | 2(8.33%) | 2 | 1(4.35%) | 1 | 2(8.33%) | 3 |
| Circulatory system | 0 | 0 | 1(4.35%) | 1 | 1(4.17%) | 1 |
| Sinus bradycardia | 0 | 0 | 1(4.35%) | 1 | 1(4.17%) | 1 |

1. **AEs after administration of 0.5 g pyrazinamide tablets in the postprandial group**

|  | Test formulation (N=24) | | Reference formulation (N=23) | | Overall (N=24) | |
| --- | --- | --- | --- | --- | --- | --- |
| Indicator | Subjects(%) | Cases | Subjects(%) | Cases | Subjects(%) | Cases |
| Laboratory examination | 2(8.33%) | 2 | 1(4.17%) | 1 | 3(12.50%) | 3 |
| Monocytosis | 1(4.17%) | 1 | 0 | 0 | 1(4.17%) | 1 |
| Urine leucocyte positive | 1(4.17%) | 1 | 1(4.17%) | 1 | 2(8.33%) | 2 |
